# A case report of dermatomyositis mimicking myasthenia gravis

**DOI:** 10.1097/MD.0000000000036234

**Published:** 2023-12-15

**Authors:** Zhang-Si Jin, Xiao-Ran Tao, Zai-Xing Wang

**Affiliations:** a Department of Dermatovenereology, the First Affiliated Hospital of Anhui Medical University, Hefei, Anhui, China.

**Keywords:** dermatomyositis, myasthenia gravis, ocular muscle weakness, thymoma

## Abstract

**Rationale:**

Patients who have myasthenia gravis or dermatomyositis show clinical signs of muscular weakening. Ocular muscle involvement is uncommon, and symmetrical proximal limb weakness is the typical presentation of dermatomyositis. However, the earliest and most noticeable sign in those with myasthenia gravis is extraocular muscular paralysis. Dermatomyositis is frequently complicated by malignancy, and the common malignancies associated with dermatomyositis vary by region and ethnicity, while thymoma is relatively rare. About 10% to 15% of people with myasthenia gravis have thymoma, which is involved in the etiology of the disease.

**Patient concerns:**

A 68-year-old female presented with ocular muscle weakness for 10 days that manifested as bilateral blepharoptosis with the phenomenon of “light in the morning and heavy in the evening.” Imaging examination showed anterior mediastinal thymic tumor with metastasis.

**Diagnoses:**

After a thorough physical examination, we discovered bilateral upper limbs with grade IV muscle strength and the typical rash of dermatomyositis. In combination with elevated serum kinase levels and electromyography suggesting myogenic damage, the patient was finally diagnosed as dermatomyositis with multiple metastases of thymoma.

**Interventions:**

The patient received oral hydroxychloroquine sulfate, topical corticosteroids, and tacrolimus ointment, but these did not work very well. Subsequently, the patient underwent surgery combined with radiotherapy for the thymoma.

**Outcomes:**

Muscle weakness in the patient improved after effective treatment of tumor, and the rash mostly disappeared.

**Conclusion:**

Ocular muscle weakness and thymoma are more common in myasthenia gravis, but we cannot ignore the possibility of dermatomyositis. To further establish the diagnosis, a thorough physical examination and laboratory findings are required. Further tumor screening should be performed for patients with dermatomyositis. Early detection and management of possible tumors are essential to the treatment of dermatomyositis linked to malignancies.

## 1. Introduction

Dermatomyositis is an idiopathic inflammatory myopathy that can involve multiple systems such as the skin, muscle, respiratory tract, digestive tract, heart, kidney and joints. Patients usually present symmetrical proximal limb muscle weakness. Characteristic skin lesions include the “heliotrope sign,” “V”-shaped erythema, “shawl sign,” and “Gottron papules.”^[1]^ In addition, dermatomyositis is frequently complicated by malignancy, and the common malignancies linked to dermatomyositis vary by geography and ethnicity.^[2,3]^ Myasthenia gravis is an autoimmune disease of acquired neuromuscular junction transmission disorder mediated by autoantibodies. Extraocular muscular paralysis is the primary symptom of myasthenia gravis in the majority of patients, who also experience the fluctuation phenomenon of “light in the morning and heavy in the evening.” The prevalence of thymoma in patients with myasthenia gravis is about 10% to 15%.^[4]^ Dermatomyositis and myasthenia gravis are both autoimmune diseases. It is crucial to distinguish between dermatomyositis and myasthenia gravis because they have similar features.^[5]^

## 2. Case report

A 68-year-old female patient was admitted to the hospital due to ocular muscle weakness for 10 days. The patient experienced bilateral blepharoptosis and diplopia with the phenomenon of “light in the morning and heavy in the evening,” mild weakness of bilateral upper limbs. She developed edematous erythema on the face, but she did not pay attention to it. The rash gradually spread to the neck, chest, and back. The patient had a history of hypertension for more than 10 years, a systolic blood pressure of up to 160 mm Hg in the past, an unknown diastolic blood pressure, and long-term oral administration of nifedipine controlled-release tablets 30 mg once daily. Mediastinal mass was found considering thymoma for 1 year. She denied any history of hepatitis, tuberculosis, diabetes, cerebrovascular disease, mental illness, trauma or blood transfusion, food or drug allergy, and denied any family history of genetic diseases or tumors. No smoking or alcohol habits.

**Figure 1.**
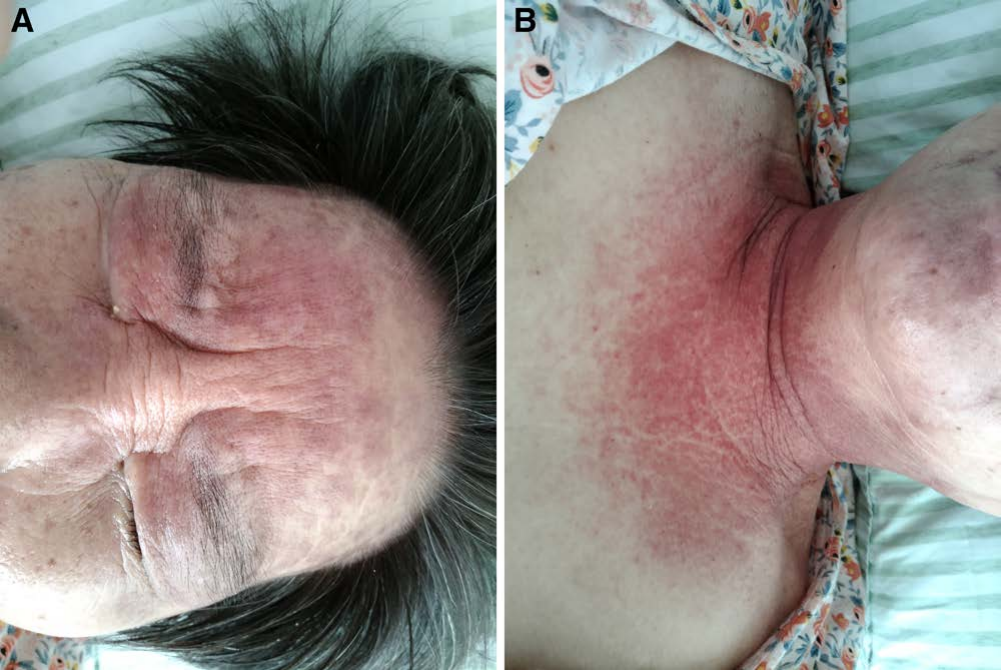
Typical rash in the patient. (A) Edematous erythema on the forehead and bilateral upper eyelids. (B) “V”-shaped erythema on anterior neck and chest.

**Figure 2.**
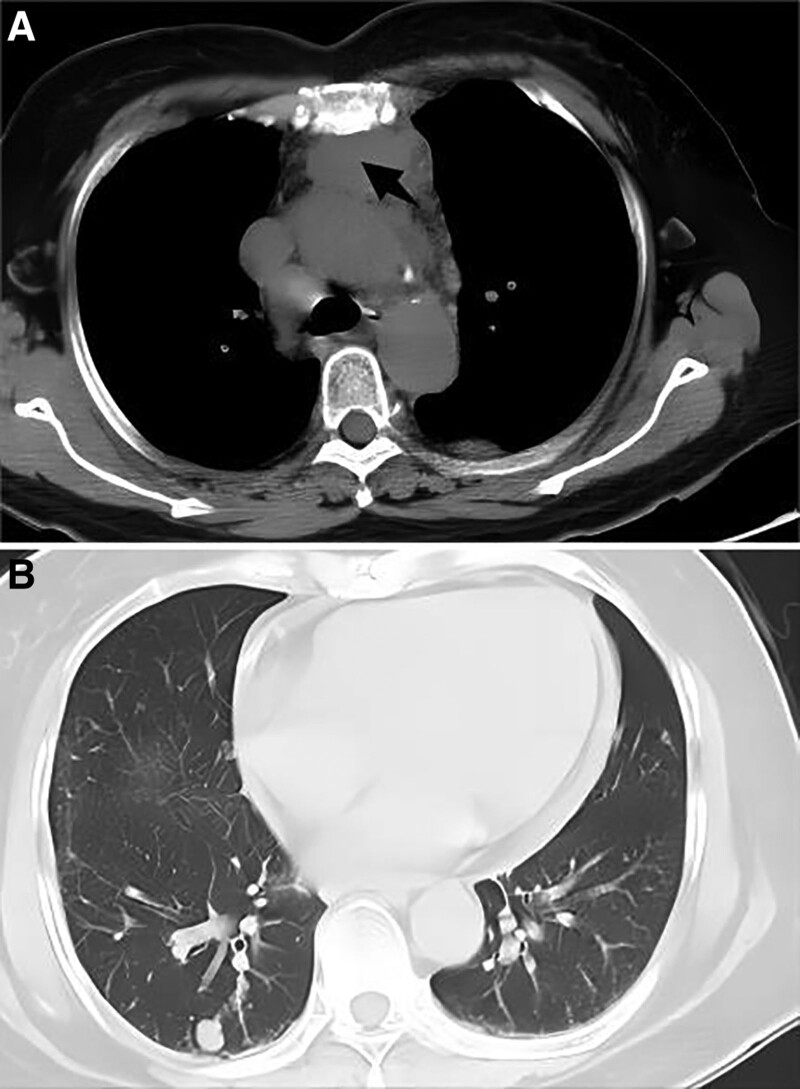
High-resolution CT image of chest. (A) Anterior mediastinal mass, considering thymoma (black arrow). (B) Multiple nodules in bilateral lung and multiple lymph nodes in mediastinum. CT = computed tomography.

**Figure 3.**
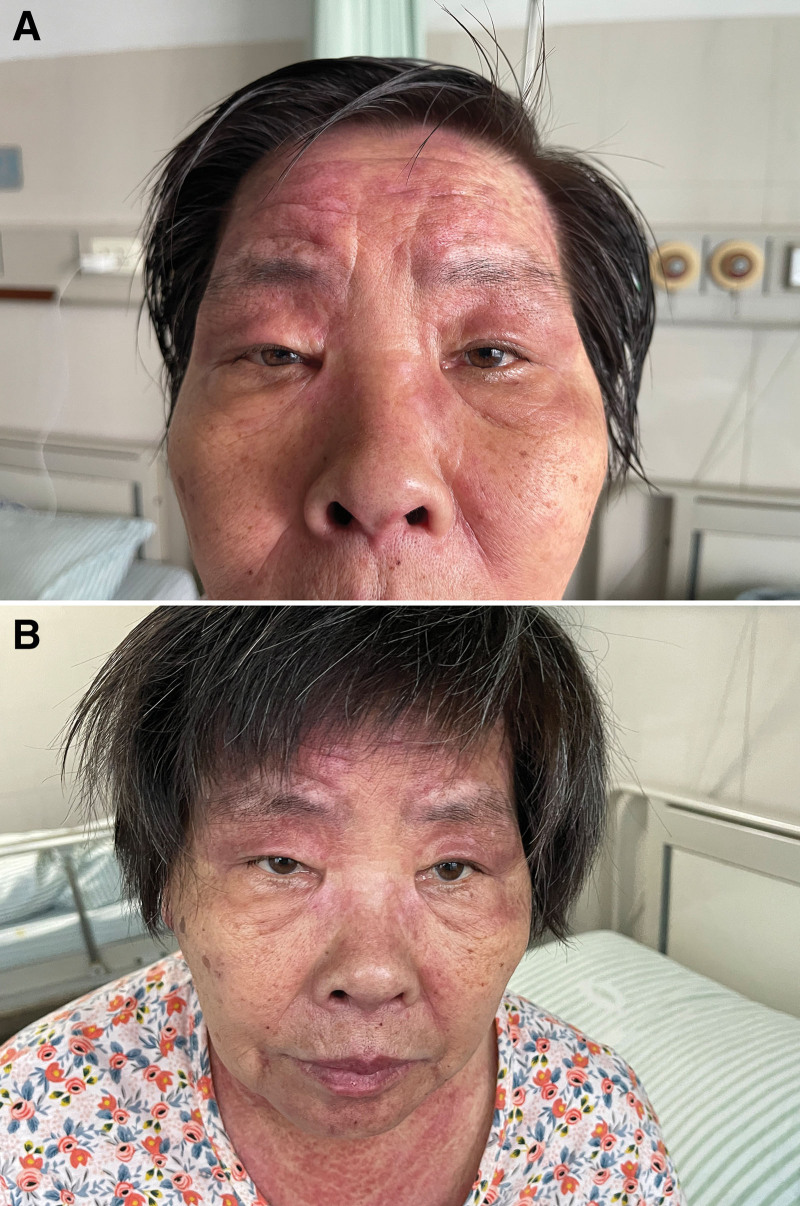
Neostigmine test was negative. Upper eyelid weakness score was measured before and 10 minutes, 20 minutes, 30 minutes, 40 minutes, 50 minutes, and 60 minutes after neostigmine injection for 7 times for 1 hour. There was no significant improvement in ocular muscle weakness symptoms. (A) Before neostigmine injection. (B) 1 hour after injection.

Specialist examination revealed edematous erythema on the forehead and bilateral upper eyelids, “V”-shaped erythema in front of the neck, and “shawl sign” on the shoulders and back (Fig. 1). Bilateral eyelids were markedly drooping and covered about 50% of the eyeball with diplopia. Muscle strength was grade IV in both upper limbs and grade V in both lower limbs, with normal muscle tone and no significant muscle tenderness throughout the body. Laboratory analyses revealed creatine kinase isoenzyme 115 U/L (reference range, 0–24 U/L), aspartate aminotransferase 90 U/L (reference range, 13–35 U/L), and lactate dehydrogenase 604 U/L (reference range, 120–250 U/L). Antinuclear antibody was positive and showed a nuclear granular pattern with a titer of 1:320. Chest computed tomography revealed an anterior mediastinal mass which was considered to be a thymic tumor. Moreover, there are multiple nodules in bilateral lung and multiple lymph nodes in mediastinum which were considered the metastases of thymoma (Fig. 2). Hepatobiliary, pancreatic, splenic, and abdominopelvic ultrasound showed multiple slightly hypoechoic lesions in the liver, also considering metastases of thymoma. Electromyography showed electrophysiological findings of myogenic Electromyography changes in the examined muscles, mainly in the proximal muscles, and compression of the bilateral median nerve wrists. Biopsy of the proximal muscle of the left upper limb revealed no significant swelling or degeneration of the muscle tissue and no significant inflammatory cell infiltration in the muscle interstitium. Neostigmine test (upper eyelid weakness score was measured before and every 10 minutes after neostigmine injection for 7 times for 1 hour) was negative (Fig. 3). Myasthenia gravis antibodies (anti-acetylcholine receptor antibody, muscle-specific receptor tyrosine kinase antibody, human low-density lipoprotein receptor-related protein 4 antibody) were also negative.

The diagnosis of dermatomyositis in the patient was definite. The patient got a combination of treatments, including an intravenous drip of vitamin C 3.0 g once daily and oral hydroxychloroquine sulfate 100 mg twice daily. Externally applying halometasone lotion to the trunk, and alternatively applying tacrolimus 0.03% ointment and hydrocortisone butyrate cream on the face. Considering that the dermatomyositis in this patient was related to tumor, it was recommended that the patient receive active tumor treatment. The patient received a call for follow-up 1 month later. In another hospital, she was given a pathological diagnosis of thymoma. The rash and muscle strength were improved after surgery combined with radiotherapy for thymoma.

## 3. Discussion

The patient had typical rash and symmetrical muscle weakness in the proximal upper limbs. Unfortunately, muscle biopsy showed no typical histopathological findings of myositis, which was thought to be due to the biopsy site or the depth at which the muscle was harvested, which did not reach the striated muscle. However, the diagnosis of dermatomyositis was established in conjunction with increased serum kinase levels and electromyography that suggested myogenic injury. It is worth noting that the patient had the prominent ocular muscle involvement and manifested as bilateral blepharoptosis with the phenomenon of “light in the morning and heavy in the evening.” The imaging examination revealed thymoma with multiple metastases. In this case, it is easy to misdiagnose as myasthenia gravis. Myasthenia gravis is an autoimmune disease of acquired neuromuscular junction transmission disorder mediated by autoantibodies, of which acetylcholine receptor antibodies are its most common pathogenic antibodies, and cholinesterase inhibitors are the drugs of choice for initial treatment. Thymoma plays a role in the pathogenesis of myasthenia gravis, and about 10% to 15% of patients have thymoma.^[4]^ Dermatomyositis with myasthenia gravis has also been documented. Dermatomyositis and myasthenia gravis are both autoimmune disorders. Muscle weakness, primarily involving striated muscles, is a defining feature of both. Extraocular muscular paralysis and the fluctuation phenomenon are the primary symptoms of myasthenia gravis in the majority of individuals. Six ocular motility muscles and 1 levator palpebrae superioris muscle, both of which are striated muscles, make up the majority of the extraocular muscles. Ptosis and diplopia, which are indicative of extraocular muscle involvement, are consistent with the symptoms of this patient.^[6–8]^ Autoimmune activation in dermatomyositis causes capillary destruction which leads to ischemia, microinfarction, hypoperfusion, and perifascicular atrophy of muscle fibers. In addition, altered expression of myogenic regulatory factors leads to impaired cell differentiation and maturation leading to muscle atrophy. Mostly, invasion of proximal limb muscle groups is the most common, and prominent extraocular muscle involvement is rare.^[9,10]^ This patient had grade IV proximal muscle strength in both of their upper limbs, and noticeable ocular muscle weakness. Neostigmine testing came out negative, and an analysis of myasthenia gravis-related antibodies revealed no abnormalities. Myasthenia gravis was not taken into account. The most crucial medications for the treatment of dermatomyositis are glucocorticoids and immunosuppressive medications. Myasthenia gravis symptoms may worsen during hormone therapy withdrawal or when high-dose hormone pulses are used when dermatomyositis is the initial condition, and even crisis may ensue. As soon as feasible, it must be determined if the patient has myasthenia gravis.^[8,11]^

Dermatomyositis is an idiopathic inflammatory myopathy which is associated with malignancy. Additionally, among connective tissue diseases, dermatomyositis has the highest frequency of malignant tumors, and these tumors are one of the most significant risk factors for death.^[1,2]^ Once dermatomyositis has been diagnosed, cancer risk evaluation and screening are required. Older age, male, experiencing dysphagia symptoms, higher creatine kinase and inflammatory markers, and having antibodies against nuclear matrix protein-2 and melanoma differentiation-associated protein-5 are all known risk factors for malignancy.^[2,3,12]^ By geography and race, different people have different common cancers linked to dermatomyositis. For instance, nasopharyngeal carcinoma is more prevalent in people in China and Southeast Asia, but breast cancer, ovarian cancer, lung cancer, and colorectal cancer are prevalent malignancies in Europe, while thymoma is relatively rare in various regions. Some scholars believe that dermatomyositis is a paraneoplastic syndrome, which can be the first or most prominent manifestation of underlying malignant tumors. This may be due to immune cross-reactivity of the tumor with normal host tissues such as skin and muscle, and thymoma is more likely to show paraneoplastic autoimmune features than other tumors.^[13]^ The general prognosis of individuals with dermatomyositis linked with malignant tumors is poor, and glucocorticoids and immunosuppressive medications are frequently inadequate. Therefore, early detection and management of potentially malignant tumors are essential to successful treatment. With the effective treatment of tumors, the symptoms and signs of dermatomyositis can be improved, while the symptoms are aggravated when the tumor progresses.^[14,15]^ Before being admitted, the patient had discovered a mediastinal lump that was perhaps a thymoma with no metastases and no evident rash or weakness of the muscles. Multiple metastases of thymoma along with obvious symptoms and signs of dermatomyositis occurred in this admission, which suggested that the progress of dermatomyositis were closely related to the tumor.

Patients with myasthenia gravis are more likely to experience noticeable ocular muscular weakening. However, dermatomyositis screening is essential and has a significant bearing on the diagnosis and course of treatment. In addition, the prognosis of patients with dermatomyositis associated with cancer is closely related to progression of tumor, and the condition of dermatomyositis can be relieved after effective anticancer therapy.

## Author contributions

**Conceptualization:** Zhang-Si Jin.

**Investigation:** Zhang-Si Jin, Xiao-Ran Tao.

**Resources:** Zai-Xing Wang.

**Supervision:** Zai-Xing Wang.

**Validation:** Zai-Xing Wang.

**Writing – original draft:** Zhang-Si Jin, Xiao-Ran Tao.

**Writing – review & editing:** Zhang-Si Jin, Zai-Xing Wang.

## References

[1] Aussy A Boyer O Cordel N. Dermatomyositis and immune-mediated necrotizing myopathies: a window on autoimmunity and cancer. Front Immunol. 2017;8:992. 28871260 10.3389/fimmu.2017.00992 PMC5566616

[2] Lim CH Tseng CW Lin CT. The clinical application of tumor markers in the screening of malignancies and interstitial lung disease of dermatomyositis/polymyositis patients: a retrospective study. SAGE Open Med. 2018;6:2050312118781895. 29977547 10.1177/2050312118781895 PMC6024348

[3] Tiniakou E Mammen AL. Idiopathic inflammatory myopathies and malignancy: a comprehensive review. Clin Rev Allergy Immunol. 2017;52:20–33. 26429706 10.1007/s12016-015-8511-x

[4] Mantegazza R Cavalcante P. Diagnosis and treatment of myasthenia gravis. Curr Opin Rheumatol. 2019;31:623–33. 31385879 10.1097/BOR.0000000000000647

[5] Yildirim F Mutlu MY İçaçan OC. Dermatomyositis associated with thymoma: a case report and literature review. Clin Ter. 2023;174:115–20. 36920126 10.7417/CT.2023.2506

[6] Shuey NH. Ocular myasthenia gravis: a review and practical guide for clinicians. Clin Exp Optom. 2022;105:205–13. 35157811 10.1080/08164622.2022.2029683

[7] Fortin E Cestari DM Weinberg DH. Ocular myasthenia gravis: an update on diagnosis and treatment. Curr Opin Ophthalmol. 2018;29:477–84. 30281029 10.1097/ICU.0000000000000526

[8] Santos E Coutinho E Martins da Silva A. Inflammatory myopathy associated with myasthenia gravis with and without thymic pathology: report of four cases and literature review. Autoimmun Rev. 2017;16:644–9. 28414153 10.1016/j.autrev.2017.04.009

[9] DeWane ME Waldman R Lu J. Dermatomyositis: clinical features and pathogenesis. J Am Acad Dermatol. 2020;82:267–81. 31279808 10.1016/j.jaad.2019.06.1309

[10] Thompson C Piguet V Choy E. The pathogenesis of dermatomyositis. Br J Dermatol. 2018;179:1256–62. 28542733 10.1111/bjd.15607

[11] Huang K Shojania K Chapman K. Concurrent inflammatory myopathy and myasthenia gravis with or without thymic pathology: a case series and literature review. Semin Arthritis Rheum. 2019;48:745–51. 29958689 10.1016/j.semarthrit.2018.05.004

[12] Yang H Peng Q Yin L. Identification of multiple cancer-associated myositis-specific autoantibodies in idiopathic inflammatory myopathies: a large longitudinal cohort study. Arthritis Res Ther. 2017;19:259. 29178913 10.1186/s13075-017-1469-8 PMC5702134

[13] Wick MR Patterson JW. Cutaneous paraneoplastic syndromes. Semin Diagn Pathol. 2019;36:211–28. 30736994 10.1053/j.semdp.2019.01.001

[14] Miranda Baleiras M Maduro L Vasques C. Paraneoplastic dermatomyositis and prostate cancer: myopathy regression under cancer-directed therapy. Dermatol Reports. 2021;13:9262. 34880970 10.4081/dr.2021.9262 PMC8611514

[15] Cobos GA Femia A Vleugels RA. Dermatomyositis: an update on diagnosis and treatment. Am J Clin Dermatol. 2020;21:339–53. 32096127 10.1007/s40257-020-00502-6

